# Necrology

**Published:** 1901-08

**Authors:** 


### NECROLOGY.

DR. LLOYD ZANER was born in Fishing Creek Township, Columbia County, April 12, 1866. The son of John and Hannah Zaner and the youngest of nine children. He obtained his preliminary education at Orangeville Academy, and graduated from the Veterinary Department of the University of Pennsylvania in 1897.

He was a faithful, quiet, earnest worker, loved by all who knew him, and was among the leading men of his class.

After graduating he began practising in Wilkesbarre, and through his ability, kindness, and industry acquired a large practice and splendid reputation.

He was married to Miss Hattie White, of Wilkesbarre, who died very suddenly, and this resulted in his complete nervous breakdown and subsequently his death.

The memory of such a man will ever be an incentive for others to follow, and the love for his profession is known by all who knew him.

